# Dr. Houston on the Mucous Membrane of the Rectum
*Dublin Hospital Reports, Vol. V.


**Published:** 1831-01-01

**Authors:** 


### DR. HOUSTON ON THE MUCOUS MEMBRANE OF THE RECTUM.*

THE structure, form, and disposition of the tunics entering into the composition of the rectum, ought, of course, to be familiar to all who venture to meddle with this gut. Yet they certainly are not so, and probably the majority of the reputed strictures of the rectum are not strictures at all, but merely some of the natural impediments offered to the introduction of foreign bodies. Dr. Houston whilst engaged in anatomical preparations illustrative of the peculiar and relative anatomy of the parts in the pelvis, discovered the existence of valves in the rectum, formed by its mucous membrane. The mode of exhibiting them consists in distending and hardening all the parts with spirit previously to being cut open.

"The valves exist equally in the young and in the aged, in the male and in the female; but in different individuals there will be found some varieties as to their number and position. Three is the average number, though sometimes four, and sometimes only two are present in a marked degree. The position of the largest and most regular valve is about three inches from the anus, opposite to the base of the bladder. The fold of next most frequent existence is placed at the upper end of the rectum. The third in order occupies a position about midway between these, and the fourth, or that most rarely present, is attached to the side of the gut, about one inch above the anus.

In addition to these valves, of tolerably regular occurrence, there are frequently several intermediate smaller ones, but which, from their trifling projection and want of regularity in their situation, merit comparatively little notice.

The form of the valves is semilunar; their convex borders are fixed to the

* Dublin Hospital Reports, Vol. V.

sides of the rectum, occupying in their attachments from one-third to one-half of the circumference of the gut. Their surfaces are sometimes horizontal, but more usually they have a slightly oblique aspect, and their concave floating margins, which are defined and sharp, are generally directed a little upwards. The breadth of the valves about their middle varies, from a half to three quarters of an inch and upwards, in the distended state of the gut. Their angles become narrow, and disappear gradually in the neighbouring membrane. Their structure consists of a duplicature of the mucous membrane, enclosing between its laminæ some cellular tissue, with a few circular muscular fibres.

The relative position of the valves, with respect to each other, deserves attention. That situated opposite to the base of the bladder, most commonly projects from the anterior wall of the gut; the valve next above from the left, and the uppermost from the right wall: that near the anus, which is of least frequent occurrence, occupies a place when present towards the left and posterior wall. Many deviations from these stated points of attachment for the folds will be found to occur, but the arrangement is nevertheless always such, as to form, by their being placed successively on different sides of the gut, a sort of spiral tract down its cavity.

In regard of the sacculated form which the rectum acquires by the presence of these valves, the gut resembles somewhat the colon in the condition of its interior; but in the peculiar spiral arrangement of the valves, it bears more an analogy to the large intestine of some of the lower animals, in which, as for example the cæcum of the rabbit, the large intestine of the serpent and dog-fish, a continuous spiral membrane traverses the cavity from end to end, and gives to the alimentary matters a protracted winding course towards the anus."

The presence of these valves may be ascertained in the unprepared body, if looked for soon after death, and before the tonic contraction of the gut has subsided. They then over-lap each other so effectually, as to require considerable manœuvre in conducting a bougie or the finger along the cavity of the intestine. Mr. Crampton uses a rectum bougie bent with a couple of light spiral turns, and in the introduction moves it about gently between his thumb and fingers. He was induced to adopt this form, from having noticed that of itself it assumed such, when allowed to become soft by remaining some time up the gut. He practices the spiral movement from observing that during its return down the canal after being thus modelled, it it disposed, if handled loosely, to take on that course. Dr. Houston suggests that these valvular folds may possibly become the most frequent seat of stricture, and he mentions some points connected with that affection which tend to support in some measure his suggestion.

